# Minimal circuit motifs for second-order conditioning in the insect mushroom body

**DOI:** 10.3389/fphys.2023.1326307

**Published:** 2024-01-10

**Authors:** Anna-Maria Jürgensen, Felix Johannes Schmitt, Martin Paul Nawrot

**Affiliations:** Computational Systems Neuroscience, Institute of Zoology, University of Cologne, Cologne, Germany

**Keywords:** associative leaning, mushroom body, second-order conditioning, classical conditioning, mechanistic model, learning and memory

## Abstract

In well-established first-order conditioning experiments, the concurrence of a sensory cue with reinforcement forms an association, allowing the cue to predict future reinforcement. In the insect mushroom body, a brain region central to learning and memory, such associations are encoded in the synapses between its intrinsic and output neurons. This process is mediated by the activity of dopaminergic neurons that encode reinforcement signals. In second-order conditioning, a new sensory cue is paired with an already established one that presumably activates dopaminergic neurons due to its predictive power of the reinforcement. We explored minimal circuit motifs in the mushroom body for their ability to support second-order conditioning using mechanistic models. We found that dopaminergic neurons can either be activated directly by the mushroom body’s intrinsic neurons or via feedback from the output neurons via several pathways. We demonstrated that the circuit motifs differ in their computational efficiency and robustness. Beyond previous research, we suggest an additional motif that relies on feedforward input of the mushroom body intrinsic neurons to dopaminergic neurons as a promising candidate for experimental evaluation. It differentiates well between trained and novel stimuli, demonstrating robust performance across a range of model parameters.

## Introduction

By forming associations between sensory cues and reinforcement during classical conditioning (first-order conditioning, FOC), animals can learn to predict the emergence of environmental factors relevant to their survival. Once a sensory cue has been established as a predictor of such reinforcement, it can act as reinforcement in second-order conditioning (SOC). SOC has been observed across species with experiments conducted in *Drosophila* (Brembs and Heisenberg, 2001; Tabone and de Belle, 2011; König et al., 2019; Rachad, 2023; Yamada et al., 2023) and other invertebrate (Bitterman et al., 1983; Hawkins, Greene, and Kandel, 1998; Hussaini et al., 2007; Mizunami et al., 2009) as well as vertebrate (Murphy and Miller, 1957; Holland and Rescorla, 1975; Cook and Mineka, 1987) species. SOC experiments involve two initially neutral stimuli (stimulus 1 and stimulus 2). Stimulus 1 is first paired directly with reinforcement during FOC, whereby it acquires a valence as a cue for reinforcement. Afterward, stimulus 2 is paired with stimulus 1 (SOC), causing an expansion of the acquired valence of stimulus 1 onto stimulus 2, without stimulus 2 itself being paired with the reinforcer. Afterward, both stimuli initiate a behavioral response based on their acquired valence.

The mushroom body (MB) is a crucial brain structure for learning and encoding relationships between sensory cues and reinforcement in *Drosophila* (De Belle and Heisenberg, 1994; Heisenberg, 1998; Zars, 2000; Heisenberg, 2003) and other insects (Mizunami, Weibrecht, and Strausfeld, 1998; Zars, 2000; Menzel and Giurfa, 2001; Rössler, 2023) alike. Its intrinsic neurons (Kenyon cells, KCs) have been shown to encode the identity of sensory inputs in *Drosophila* (Wang et al., 2004; Turner, Bazhenov, and Laurent, 2008; Honegger, Campbell, and Turner, 2011; Lin et al., 2014) and other insects (Perez-Orive et al., 2002; Stopfer, Jayaraman, and Laurent, 2003; Szyszka et al., 2005; Demmer and Kloppenburg, 2009). Across species, they relay their output onto a much smaller number of MB output neurons (MBONs) (Rybak and Menzel, 1993; Li and Strausfeld, 1997; Fahrbach, 2006; Tanaka, Tanimoto, and Ito, 2008; Aso et al., 2014a; Strube-Bloss and Rössler, 2018). Plasticity at the synapses from KCs to MBONs (KC>MBON) allows MBONs to encode the learned valence of a sensory cue, according to extensive experimental evidence obtained in *Drosophila* (Strube-Bloss, Nawrot, and Menzel, 2011; Aso et al., 2014b; Hige et al., 2015; Owald et al., 2015; Owald and Waddell, 2015; Barnstedt et al., 2016). Neuromodulators, such as dopamine mediate this plasticity (Schwaerzel et al., 2003; Waddell, 2010; Kim et al., 2013; Hige et al., 2015; Aso and Rubin, 2016; Sachse and Beshel, 2016; Mizunami and Matsumoto, 2017). In *Drosophila*, it has been shown that either an inherently punishing or rewarding stimulus (electric shock, sugar) (Tabone and de Belle, 2011; Rachad, 2023; Yamada et al., 2023) or direct optogenetic activation of dopaminergic neurons (DANs) (Yamada et al., 2023) can be utilized to deliver a reinforcement signal during FOC phase of such experiments to establish second-order memory later. Experiments in *Drosophila* have demonstrated that stimulus 1 itself causes activation of DANs and enhances it after being paired with reinforcement (Riemensperger et al., 2005; Mao and Davis, 2009; Dylla et al., 2017; Rachad, 2023; Yamada et al., 2023). The mechanism inducing synaptic plasticity, not only during FOC but also during SOC, likely relies on DAN activation. During FOC, reward or punishment mediating DANs are activated directly by the reward or punishment and indirectly by the altered network response to stimulus 1 during SOC. The strength of the behaviorally expressed stimulus 1 and stimulus 2 valence after SOC can be similar (Tabone and de Belle, 2011; Yamada et al., 2023) or weaker (Rachad, 2023; Yamada et al., 2023) for stimulus 2.

Two different circuit mechanisms lend themselves to achieving such post-FOC activation of DANs by stimulus 1: Firstly, a stimulus 1 representation among the KCs could serve as direct feed-forward stimulus-specific input to the DANs (Cervantes-Sandoval et al., 2017; Dylla et al., 2017; Eichler et al., 2017; Takemura et al., 2017; Saumweber et al., 2018; Eschbach et al., 2020; Scheffer et al., 2020). Alternatively, the input could be supplied via MBON feedback (Ichinose et al., 2015; Eichler et al., 2017; Takemura et al., 2017; Eschbach et al., 2020). Their response to stimulus 1, altered by learning, could serve as a manifestation of stimulus-specific valence.

Here, we tested possible circuit motifs that could underlie SOC in the insect MB using abstract and simplified network models inspired by the *Drosophila* olfactory pathway and the MB. For simplicity, we focus on a reward learning paradigm that could be adapted to punishment learning by introducing an additional DAN for the encoding of a respective negative reinforcement signal. Starting from a basic model of the MB, we explored different circuit configurations and their capacity to produce SOC in an olfactory learning protocol to identify promising candidates for experimental testing. To define our solution space, we assumed that learning in the MB depends on KC>MBON plasticity, mediated by a dopamine signal during both FOC and SOC. Model circuits should be able to produce both FOC and SOC without generalizing associations with reinforcement unspecifically to novel stimuli. We tested all models in classical conditioning experiments and demonstrated their ability to support FOC and SOC. Additionally, we evaluated differences in their biological plausibility by quantifying robustness and discussing functional and anatomical evidence for the respective circuits. We found that a particular circuit that achieves DAN activation through plastic excitatory KC input during SOC outperforms the other candidates and appears compatible with the MB anatomy. We suggest this circuit motif, which differs from previously reported mechanisms (König et al., 2019; Rachad, 2023; Yamada et al., 2023), as an additional, novel candidate for experimental tests.

## Materials and methods

**FIGURE 1:**
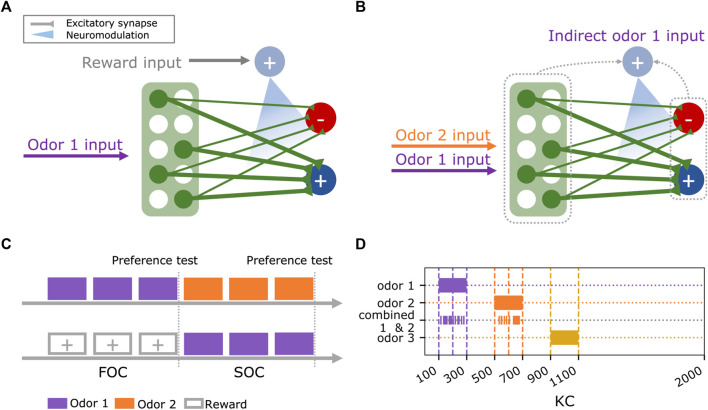
Experimental design for testing second-order conditioning. **(A)** Basic circuit motif for first-order conditioning, consisting of 2000 Kenyon cells (green), two output neurons (dark blue, red), and a single dopaminergic neuron (light blue). The co-occurrence of odor and reward input elicits plasticity at the mushroom body output synapses. **(B)** During second-order conditioning, the dopaminergic neuron (light blue) is indirectly activated by the previously trained odor 1 and paired with odor 2. We test different candidate mechanisms for this indirect activation of the dopaminergic neuron via the Kenyon cells (green) or the mushroom body output neurons (dark blue, red). **(C)** The experimental paradigm consists of two phases (first and second-order conditioning, FOC and SOC). During first-order conditioning, odor 1 is paired with a reward. Subsequently, a novel odor 2 is paired with odor 1 during second-order conditioning. Odor valences are tested after first and second-order conditioning. **(D)** Initially, three non-overlapping odors were used in the experiments. Odors are encoded as Kenyon cell activity patterns. The joint presentation of odor 1 and odor 2 during second-order conditioning retains a randomly chosen 50% of the individual odor representations to maintain the same overall activation as with individually presented odors.

The basic model of a minimal circuit motif in the MB for FOC (Figure 1A) was extended with different mechanisms for SOC (Figure 1B) and trained and tested in simulated classical conditioning experiments (Figure 1C) using three different odors (Figure 1D).

### Network input

Odor and reward signals were provided to all models via the KCs and the DAN (Figures 1A, B), respectively. Three odors were used in the experiments. In the most simple case, each exclusively activates an independent combination of (10%) of the 2000 KCs (Figure 1D) with a rate of 3 Hz to match the levels of population sparseness and low odor-response rates reported in KCs (Perez-Orive et al., 2002; Ito et al., 2008; Turner, Bazhenov, and Laurent, 2008). The first experiment combined the three odors. For each model instance, odor 1 activates a combination of 200 randomly chosen KCs. KCs activated by odor 2 and odor 3 are then sequentially drawn from the combination of remaining KCs. When odors 1 and 2 were presented together during the experiments, each component of this compound odor activated a random 50% of the KCs, activated by each of the individual odors. This ensured the proportion of activated KC would not exceed 10% (Honegger, Campbell, and Turner, 2011).

A peculiarity for the quantification of model SOC performance arises from this implementation of compound odors. During SOC, odors 1 and 2 are presented as a compound, activating 200 KCs in total (50% of odor 1% and 50% of odor 2 activated KCs). We quantified the approach bias (Eq. 1) for odors 1 and 2 as a measure of learning during FOC and SOC, respectively. This measure is based on the ratio between the two approach and avoidance MBON (MBON^+^, MBON^−^) output rates *R*. The model parameters were optimized in a way that guaranteed a perfect approach bias for odor 1 after FOC (see Results: Identifying optimal parameters for each model). Mathematically, this entails that the 200 KC>MBON^−^ weights altered for odor 1 during FOC will be at 0. In comparison, only 100 KC>MBON^−^ weights targeted by odor 2 will be altered during SOC due to the compound odor activation pattern. Given inital KC>MBON weights of 0.083 (Table 1) and a KC odor response of 3 Hz (Table 1), this yielded an approach bias (Eq. 1) of (49.8–0)/(49.8 + 0) for odor 1 after FOC and (49.8–24.9)/(49.8 + 24.9) for odor 2 after SOC. In all equations, $\vec{x}$ always denotes a vector. *R* represents the activity of a neuron, which can be interpreted as a spike rate of a neuron or a vector of neurons in the case of KCs, and *w* denotes a synaptic weight or a vector of weights.

$$B=\frac{R_{MBON^{+}}-R_{MBON^{-}}}{R_{MBON^{+}}+R_{MBON^{-}}}$$ (1)

**TABLE 1:** Fixed parameters shared between models.

Parameter	Value
Seed	999
trials_FOC	3
trials_SOC	3
num_KC	2000
*KC*_*baseline*_	0 Hz
odor_activation	3 Hz
odor_FOC	odor 1
odor_SOC	odor 1_2
$init_{KC>MBON^{+}}$	0.083
$init_{KC>MBON^{-}}$	0.083

An additional experiment was conducted as a control where the KC activation patterns for odors 1 and 2 were added during SOC (yielding 400 active KCs). The maximal performance achieved by models 2-5 was 1 in this case.

The second experiment aimed to quantify the stability of the different circuit mechanisms against the generalization of the learned valence onto novel odors. Therefore, a varying degree of odor similarity of odor 3 with odor 1 or odor 2 was used. Odor similarity is implemented as an overlap in activated KCs for odor 1 and odor 2. If any given odor activates 200 KCs, an odor similarity of 50% would yield odors 1 and 2 to have an overlap of 100 KCs. During the joint presentation of both odors, 150 KCs would be activated.

### Parameter optimization

Some parameters were fixed to a specific value (Table 1) across all models (Table 2, see Results: Candidate circuits for second-order conditioning). The remaining parameters were optimized for FOC and SOC performance using a grid search, if possible, within the same boundaries and with the same step size (Table 3). Some parameters were fixed to the same value for all models to adhere to biological constraints (Table 1). KCs show very little spontaneous activity (Perez-Orive et al., 2002; Ito et al., 2008; Turner, Bazhenov, and Laurent, 2008) and sparse activation (Perez-Orive et al., 2002; Ito et al., 2008; Turner, Bazhenov, and Laurent, 2008; Vrontou et al., 2021). MBON rates of up to 40 Hz have been shown for one MBON (Hige et al., 2015). We chose the initial weights for all KC>MBON synapses to achieve plausible MBON rates of no higher than 50 Hz. The upper limit of the DAN rate was 20 Hz to match the experimental literature (Huang et al., 2022).

**TABLE 2:** Equations underlying the different models.

**Model 1**	$R_{\mathrm{DAN}}\left(t\right)=Ip_{\mathrm{ext}}\left(t\right)+{\vec{R}}_{KC}\left(t\right)\cdot {\vec{w}}_{KC>DAN}\left(t\right)\;\left(8\right)$
	$w_{KC>KC}^{ij}\left(t+1\right)=\left\{\begin{array}{cc} w_{KC>KC}^{ij}\left(t\right)+\alpha_{KC>KC},\; & \text{if }\;R_{KC}^{i}\left(t\right)>KC_{\mathrm{baseline}}\text{ and}\\ \; & R_{KC}^{j}\left(t\right)>KC_{\mathrm{baseline}}\\ w_{KC>KC}^{ij}\left(t\right),\; & \text{otherwise} \end{array}\right.\;\left(9\right)$
**Model 2**	$R_{\mathrm{DAN}}\left(t\right)=Ip_{\mathrm{ext}}\left(t\right)+{\vec{R}}_{KC}\left(t\right)\cdot {\vec{w}}_{KC>DAN}\left(t\right)\;\left(10\right)$
	$w_{KC>DAN}^{i}\left(t+1\right)=\left\{\begin{array}{cc} w_{KC>DAN}^{i}\left(t\right)+\alpha_{KC>DAN}\cdot R_{\mathrm{DAN}}\left(t\right),\; & \text{if }\;R_{KC}^{i}\left(t\right)>0\\ w_{KC>DAN}^{i}\left(t\right),\; & \text{otherwise} \end{array}\right.\;\left(11\right)$
**Model 3**	$R_{\mathrm{DAN}}\left(t\right)=Ip_{\mathrm{ext}}\left(t\right)+R_{MBON^{+}}\left(t\right)\cdot w_{MBON^{+}>DAN}\left(t\right)-R_{MBON^{-}}\left(t\right)\cdot w_{MBON^{-}>DAN}\left(t\right)\;\left(12\right)$
**Model 4**	$R_{\mathrm{DAN}}\left(t\right)=Ip_{\mathrm{ext}}\left(t\right)+R_{\mathrm{DANbaseline}}-R_{MBON^{-}}\left(t\right)\cdot w_{MBON^{-}>DAN}\left(t\right)\;\left(13\right)$
**Model 5**	$R_{\mathrm{DAN}}\left(t\right)=Ip_{\mathrm{ext}}\left(t\right)+R_{MBON^{+}}\left(t\right)\cdot {\vec{w}}_{MBON^{+}>DAN}\left(t\right)\;\left(14\right)$
	${\vec{w}}_{MBON^{+}>DAN}\left(t+1\right)=\left\{\begin{array}{cc} {\vec{w}}_{MBON^{+}>DAN}\left(t\right)+\alpha_{MBON^{+}>DAN}\cdot R_{\mathrm{DAN}}\left(t\right),\; & \text{if }\;R_{MBON_{+}}\left(t\right)>0\\ {\vec{w}}_{MBON^{+}>DAN}\left(t\right),\; & \text{otherwise} \end{array}\right.\;\left(15\right)$

**TABLE 3:** Optimized model parameters. For all models, 100 equally distributed values per parameter between the minimum and maximum values were used to construct a regular grid of parameter combinations.

Parameter	Min	Max	Optimum
**Model 1**			
*init*_*KC*>*KC*_	0	0	0
*init*_*KC*>*DAN*_	0.0	0.001	0.001
$\alpha_{KC>MBON^{-}}$	0.001	0.004	0.004
*α*_*KC*>*KC*_	0.0	0.004	0.000162
DAN_activation	1.0	10.0	7.272,727
**Model 2**			
*init*_*KC*>*DAN*_	0.0	0.001	0.000505
$\alpha_{KC>MBON^{-}}$	0.001	0.004	0.003333
*α*_*KC*>*DAN*_	0.0	0.001	0.000677
DAN_activation	1.0	10.0	5.727,273
**Model 3**			
$init_{MBON^{+}>DAN}$	0.0	1.0	0.272,727
$init_{MBON^{-}>DAN}$	0.0	1.0	0.262,626
$\alpha_{KC>MBON^{-}}$	0.001	0.004	0.003121
DAN_activation	1.0	10.0	5.00
**Model 4**			
$init_{MBON^{-}>DAN}$	0.0	0.5	0.131,313
$\alpha_{KC>MBON^{-}}$	0.001	0.004	0.003182
*R*_*DANbaseline*_	0.0	30.0	11.212,121
DAN_activation	1.0	10.0	3.727,273
**Model 5**			
$init_{MBON^{+}>DAN}$	0.0	0.4	0.080808
$\alpha_{KC>MBON^{-}}$	0.001	0.004	0.003182
$\alpha_{MBON^{+}>DAN}$	0.001	0.01	0.003
DAN_activation	1.0	10.0	4.727,273

A grid search was conducted for each model to optimize the free model parameters (Table 3) for FOC and SOC while minimizing reward generalization to novel odors (see Results: Identifying optimal parameters for each model). All searches contained 100^4^ = 100 ⋅ 10^6^ parameter combinations for testing (Table 3). The grid searches for all models were run on the same server (X86_64 architecture, Ubuntu 20.04.3). The simulation of the parameter combinations was distributed across 24 independent processes using the same random seed. The resulting data were first filtered for the fulfillment of the rate criteria for MBONs and the DAN and the performance in the FOC and SOC tests to determine all equally good parameter combinations, which we term *optimal learners*. Grid search for all models 1-5 yielded several *optimal learners*. We computed the average *optimal learner* by averaging all *optimal learners* within every parameter. We argue that this average *optimal learner* approximates the center of all equally good parameter combinations. Next, Euclidean distances were computed between all z-standardized *optimal learners* and the average *optimal learner*. The parameter combination with the smallest distance to the average parameter combination in an n-dimensional z-standardized space (*n* = number of optimized parameters) was selected as the most central *optimal learner* (Table 3). We assume that parameter combinations closer to the average can be considered biologically more plausible because their central location makes them more robust to parameter deviations in all directions (see Discussion).

### Hypersphere sampling

To compare the robustness in a larger parameter space between the different models, we used a sphere with an increasing radius around a central point to sample parameter combinations and evaluate their effect on the respective model’s learning performance. We positioned a 4-dimensional hypersphere (Krauth, 2006) with radius *r* around the most central *optimal learner* (Table 3). For each model, we increased *r* of the hypersphere in 100 linearly spaced steps from 0 to 1 in a standardized space for easier comparison between the different models. Standardization was done by multiplying each coordinate of the 4-dimensional points with the range (max-min, Table 3) for the respective parameter and model and adding the initial position (central *optimal learner*) to it.

We then sampled 700 points **x** uniformly from the surface of the 4-dimensional hypersphere for each radius *r* by drawing all four components independently of Gaussian distributions with a standard deviation *σ* and scaling with the norm ‖**x**‖ (Eq. 2):

$$\begin{array}{cc} \sigma & \leftarrow 1\\ \vec{x} & \leftarrow \left[\text{Gauss}\left(\sigma \right),\text{Gauss}\left(\sigma \right),\text{Gauss}\left(\sigma \right),\text{Gauss}\left(\sigma \right)\right]\\ \vec{x} & \leftarrow r\cdot \frac{\vec{x}}{\left\|\vec{x}\right\|}. \end{array}$$ (2)

Each sample was evaluated by an indicator function (Eq. 3):

$$1_{O}\left(\vec{x}\right)≔\left\{\begin{array}{cc} 1\;\; & \text{ if }\;\vec{x}\in O\;\text{optimal set, optimal performance,}\\ 0\;\; & \text{ if }\;\vec{x}\notin O. \end{array}\right.$$ (3)

A parameter combination $\vec{x}$ is an element of the set of *optimal learners* *O* if it shows the same optimal performance in the FOC and SOC tests as the most central *optimal learner* for the specific model. The code for implementing the circuit models can be obtained at https://github.com/nawrotlab/exploring_SOC_circuits.

## Results

### First-order conditioning in a basic mushroom body circuit

The basic network consists of 2000 KCs, each innervating two MBONs (Figure 1A) and a single reward-mediating DAN. Each neuron is characterized by a rate representing the activation of a single neuron. It has been shown that MBONs receive inputs from many of the KCs in adult *Drosophila* (Aso et al., 2014a; Takemura et al., 2017). For simplicity, we started by modeling complete KC>MBON connectivity. Some MBONs can be categorized as approach or avoidance signaling (Séjourné et al., 2011; Aso et al., 2014b; Hige et al., 2015; Owald et al., 2015). In the model, this corresponds to MBON^+^ (Eq. 4) and MBON^−^ (Eq. 5), respectively. Other types of MBONs were disregarded here. Initially, all synaptic KC>MBON weights were set to the same value (Table 1). The DAN can be activated by the external input, representing a reinforcer in the environment (Figure 1A, Eq. 6). In a reward learning experiment, KC>MBON^−^ synapses undergo plasticity whenever trial-based KC activation, driven by odor input, and DAN activation coincide. We employ a two-factor learning rule (Eq. 7) at the KC>MBON^−^ synapses, leading to a decrease in the synaptic weights with the limitation that they can not take on a negative value. DAN activation (Eq. 6) is the sum of the model-specific external input rate *Ip*_*ext*_ (Table 2) that represents reinforcement and the network internal input *Ip*_*int*_, provided via the different circuit mechanisms. In all equations *α* refers to the learning rate at the KC>MBON^−^ synapses (Table 1).

$$R_{MBON^{+}}\left(t\right)={\vec{R}}_{KC}\left(t\right)\cdot {\vec{w}}_{KC>MBON^{+}}\left(t\right)$$ (4)

$$R_{MBON^{-}}\left(t\right)={\vec{R}}_{KC}\left(t\right)\cdot {\vec{w}}_{KC>MBON^{-}}\left(t\right)$$ (5)

$$R_{\mathrm{DAN}}\left(t\right)=Ip_{\mathrm{ext}}\left(t\right)+Ip_{\mathrm{int}}\left(t\right)$$ (6)

$$w_{KC>MBON^{-}}^{i}\left(t+1\right)=\left\{\begin{array}{cc} w_{KC>MBON^{-}}^{i}\left(t\right)-\alpha \cdot R_{\mathrm{DAN}}\left(t\right),\; & \text{if}\;R_{KC}^{i}\left(t\right)>0\text{ and }\\ \; & w_{KC>MBON^{-}}^{i}\left(t\right)>\left(\alpha \cdot R_{\mathrm{DAN}}\left(t\right)\right)\\ w_{KC>MBON^{-}}^{i}\left(t\right),\; & \text{otherwise} \end{array}\right.$$ (7)

### Candidate circuits for second-order conditioning

**FIGURE 2:**
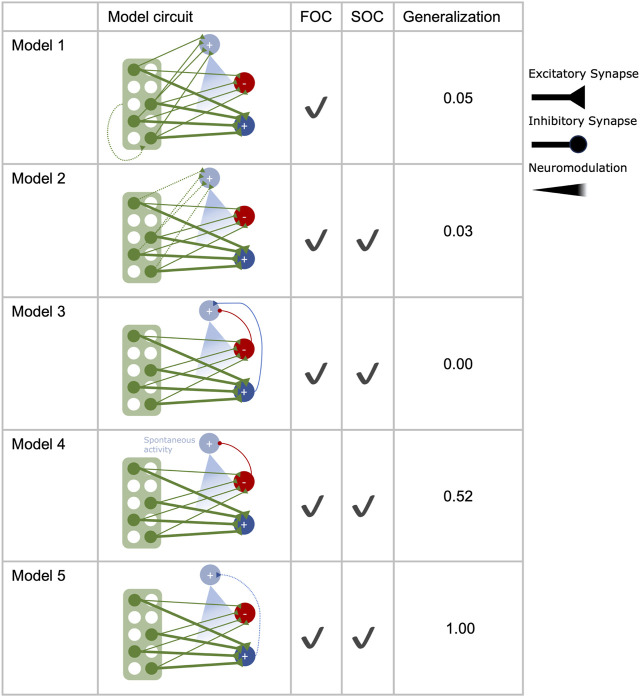
Second-order conditioning in different circuit motifs. Five different circuits were tested for their performance in first (FOC) and second-order conditioning (SOC) and the extent to which the odor-reward association generalizes to another novel odor. All circuits are constructed with 2000 Kenyon cells (green), two mushroom body output neurons (dark blue, red), and a single dopaminergic neuron (light blue), targeting the synapses between Kenyon cells and mushroom body output neurons. Additional feed-forward connections from the Kenyon cells (model 1, model 2) or feedback connections from the mushroom body output neurons onto the dopaminergic neuron (model 3, model 4, model 5) are implemented in the different circuits.

Using the basic circuit model (Figure 1A) as a starting point, we implemented five different extended versions of it (Figure 2). These models either rely on some form of KC>DAN input (model 1, model 2) or MBON>DAN feedback (model 3, model 4, model 5) as a means to expand the learned association of odor 1 with reinforcement to odor 2 during SOC. Unless specified otherwise, the DAN is not spontaneously active. The equations for all models can be found in Table 2.

To compare the different circuit motifs in an unbiased manner, their parameters were optimized using a grid search (see Materials and methods), except the fixed parameters shared between all models (Table 1), which were kept constant to allow better comparison between the candidate mechanisms for SOC implemented in the different circuits. The goal for parameter optimization was to identify parameter combinations for each model that yield the best learning performance in an associative learning experiment that consisted of a combination of FOC and SOC learning trials (Figure 1B). In insect learning experiments, forming a direct or indirect association with reward leads to approach behavior that can manifest in the movement toward the source of an odor or feeding-related behavior (Bitterman et al., 1983; Hussaini et al., 2007; Yamada et al., 2023). In our model experiments, the successful acquisition of an association with reward was quantified using the approach bias (Eq. 1) because MBON activity has been shown to initiate approach or avoidance behavior (Aso et al., 2014b; Owald et al., 2015; Hancock et al., 2022).

Model 1
  Model 1 includes KC>DAN synapses of a fixed strength (Eq. 8) and plastic KC>KC excitatory feedback with increments of *α*_*KC*>*KC*_ (Table 3), triggered by pre and post-synaptic KC activation (Eq. 9).
Model 2
  Model 2 expands the basic circuit with excitatory plastic synapses between KCs and the DAN (Eq. 10). They are each initialized with *init*_*KC*>*DAN*_ (Table 3) and are increased by *α*_*KC*>*DAN*_ (Table 3) when activation of the respective KC coincides with DAN activity, yielding DAN activation (Eq. 11).
Model 3
  In model 3, network input into the DAN is implemented via feedback from both MBONs (Eq. 12). Inhibitory input with a fixed synaptic strength comes from MBON^−^, while excitatory input is provided by the MBON^+^.
Model 4
  Model 4 uses a spontaneously active DAN (*R*_*DANbaseline*_, (Table 3) in combination with inhibitory MBON^−^>DAN input (Eq. 13). Both effects regulate the DAN activation in the absence of reward.
Model 5
  In model 5, an excitatory plastic MBON^+^>DAN synapse is added to the basic circuit (Eqn. 14). When MBON^+^ and DAN activity coincide, the synaptic strength is increased by $\alpha_{MBON_{+}>DAN}$ (Table 3) for each DAN spike (Eq. 15). During FOC, this synapse is strengthened, allowing for activation of the DAN during SOC. This allows KCs to activate the DAN.

### Optimal model parameters

All models were trained and tested in a combined FOC and SOC protocol, where odors were used as stimuli and tested for their approach bias first after completing FOC with odor 1 and reward, then after completing SOC with odors 1 and 2 (Figure 1B; Eq. 1). Plasticity was disabled during testing to isolate odor valence acquired during the respective training trials without the influence of the test itself. Additionally, a novel odor 3 was included in both tests to examine any generalization of the reward association that might have occurred during the FOC or SOC training processes. To assess the ability of the different circuit motifs to support SOC, we optimized each model for the highest possible SOC performance, which translates to maximizing the approach bias for odor 2 after three SOC trials. Additionally, we introduce several criteria the models must fulfill to ensure that the learning effect for odor 2 is odor-specific and originates from the respective mechanism applied during the SOC trials. These criteria are:

• post FOC odor 1 approach bias = 1
• post FOC odor 2 approach bias = 0
• post FOC odor 3 approach bias = 0
• post SOC odor 1 approach bias = 1
• post SOC odor 3 approach bias = 0

Additionally, DAN and MBON rates should never exceed 20 Hz and 50 Hz, respectively, to stay within the biologically realistic range for MBONs (Hige et al., 2015) and DANs (Huang et al., 2022). Among all parameter combinations that fulfilled these criteria (*optimal learners*), we selected the most centrally located one (see Materials and methods: Parameter optimization), which we refer to as the most central *optimal learner*.

**FIGURE 3:**
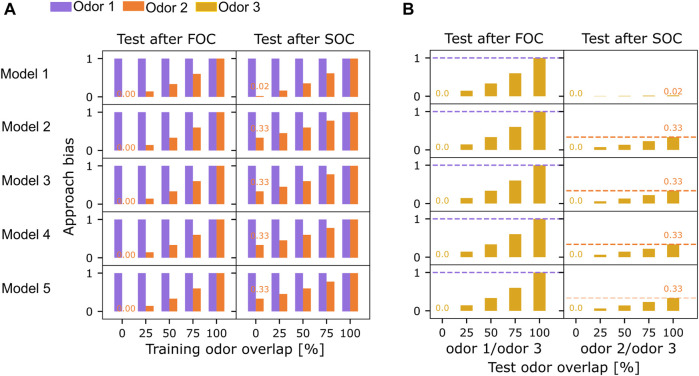
Reward generalization for overlapping training or test odors. **(A)** All five models were tested for their odor approach bias (Eq. 1) to odors 1 (purple) and 2 (orange) after first (FOC) and second-order conditioning (SOC). The overlap between odors 1 and 2 was varied. **(B)** All models were tested for their approach bias (Eq. 1) to odor 3 after training with non-overlapping odors 1 and 2. Overlap between odors 3 and 1 or 2 was varied, respectively. Orange and purple dashed lines indicate each model’s FOC and SOC performance from an experiment without odor overlap as a reference (always the first bar).

The basic learning model (Figure 1A) fulfilled the criteria for FOC learning, but no parameter combination could accommodate SOC, yielding no approach bias for odor 2 after SOC. There was at least one optimal parameter combination that fulfilled the optimization criteria for each extended candidate circuit (Figures 2, 3A ). All models acquired an optimal approach bias of 1 for odor 1 at both test times, indicating that the association of odor 1 and reward is learned during FOC and fully retained throughout the SOC protocol. Tests with odor 3 always yielded an approach bias of 0 for all models, indicating that the approach bias does not generalize inadmissibly to novel and fully disjunct odors. All models, except model 1, achieved equally good SOC performance, as indicated by an approach bias of 0.33 for odor 2 after SOC. Model 1 only acquired a bias of 0.02. For each model, the maximum value of SOC performance is determined by the implementation of the compound presentation of odors 1 and 2 (see Materials and methods). The approach bias of 0.33 for SOC (Eq. 1) is the highest value any model can achieve in this experiment (see Materials and methods: Network Input). In none of the models, any approach bias for the disjunct, novel odor 3 was observed. Additionally, we extended the experimental protocol (Figure 1B) with three trials of presenting odor 3 alone and without any reward after SOC and included another test. Depending on the degree of specificity with which the different model circuits activate the DAN, unwanted generalization of the valence to odor 3 was observed. Models 1-3 outperformed models 4 and 5 here (Figure 2).

### Learning generalizes with increasing odor overlap

The overlap of odors 1 and 2 during training and testing was varied separately (0%, 25%, 50%, 75%, 100% overlap), encoded in the percentage of KCs jointly activated between the individually presented odor 1 and odor 2 (see Materials and methods: Network input). Across all models, both FOC and SOC approach biases (Eq. 1) increase with the overlap between odor 1 and odor 2 (Figure 3A). Between highly overlapping odors, the reward association generalizes. This results in an approach bias for odor 2 after FOC, even though odor 2 was not presented during FOC (Figure 3A). A joint presentation of odor 1 and odor 2 during SOC then leads to an even higher approach bias for odor 2 (Figure 3A). All models acquire similar biases (Eq. 1) for odor 2, depending on the degree of overlap, except model 1, where the SOC learning effect was always lower (Figure 3A).

Keeping odor 1 and odor 2 fully disjunct, we varied the degree of overlap between odor 3 and either odor 1 (Figure 3B, left) or odor 2 (Figure 3B, right) during both tests. For all models, the approach bias for odor 3 after FOC scaled with the overlap and reached the same value as odor 1 if fully overlapping (Figure 3B, dashed purple lines). Testing again after SOC yielded the same results, which are not depicted here. When the overlap between odor 3 and odor 2 varied at both test times, no approach bias was observed after FOC since odor 2 was not presented during the FOC trials (results not shown). In a test after SOC, all models perform similarly concerning the upper bound of the approach bias at the magnitude reached by odor 2 (Figure 3B, dashed orange lines).

### Robustness of second-order conditioning varies across the different model circuits

In the conditioning experiments reported thus far, all model circuits, except model 1, performed equally well (Figures 2, 3). To further differentiate between them, we next examined the robustness of the model’s performances to variations of their parameters using three different methods.

We first quantified the percentage of *optimal learner* parameter combinations within the searched parameter grid for each model. A real brain would likely not require a single, extremely precise combination of physiological parameters to perform any computational task, such as SOC. Since the parameters of our computational models are ultimately representations of neuronal or synaptic characteristics, the stability of SOC performance across parameter combinations could hint at the degree to which a circuit motif is biologically plausible. For each respective model, four parameters were optimized, yielding a grid with 100^4^ parameter combinations. In the case of model 1, 5.9^–5^% of parameter combinations were equally *optimal learners*. Model 2 yielded 6.81%, model 3 only 0.37%, model 4 2.11% and model 5 4.29% *optimal learner* parameter combinations. From this perspective, models 2, 4, and 5 thus seem more robust compared to models 1 and 3.

**FIGURE 4:**
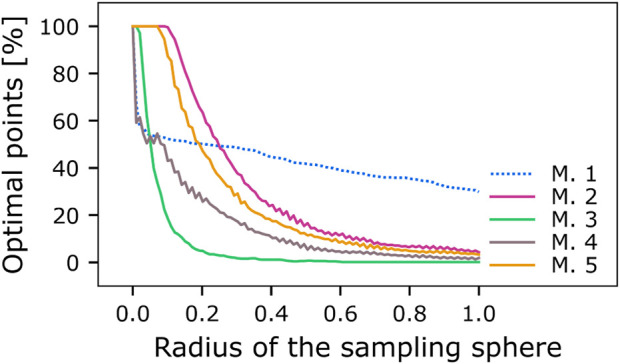
Comparison of parameter robustness between models. The five models (M.1-M.5) were tested for the stability of their second-order learning performance (Eq. 1) when their optimal parameter combinations were collectively shifted away from their most central *optimal learner*, which we used as the starting point for a hypersphere with radius = 0. We incrementally increased the radius of the hypersphere from 0 to 1 with 100 steps in a linear fashion. We sampled 700 data points from its surface for each resulting radius in a standardized space. Standardization was done using the minimum and maximum parameters tested and centered using the most central *optimal learner* (Table 3). Each sampled parameter combination was evaluated for optimal SOC performance of 0.33 (models 2–5) or 0.02 (model 1).

As an additional measure to assessing the optimal portion of the entire solution space of possible parameter combinations, we used a method for individually sampling the four-dimensional Euclidean parameter space for each model. The four-dimensional space for each model was standardized using the range of the grid (max-min parameter value, Table 3). A four-dimensional hypersphere was positioned as the point representing most central *optimal learner*, with radius = 0. We then incrementally increased the radius of the hypersphere from 0 to 1 in the standardized space with 100 linearly spaced steps and, for each increase, sampled 700 data points from its surface (see Materials and methods, Hypersphere sampling). These sampled data points do not necessarily correspond to data points from the set of equally optimal parameter combinations found in the grid search for the respective model due to the step size used in the parameter optimization. The sampled points were transformed back into their original space and then used to simulate the respective model to examine if this parameter combination would yield FOC and SOC performance that fit the criteria for the *optimal learner* for the respective model (optimal SOC performance differs between models two to five and model 1 with 0.33 and 0.02, respectively, Figure 3). For each radius increment, we calculated the percentage of sampled points from the hypersphere surface that exhibit this optimal performance (Figure 4). Since the parameter space was standardized for each model and the radii used were the same, the results can easily be compared between models 2–5. We find that the models differ in their robustness to deviations of the parameters from their optimal values. Models 2 and 5 strongly outperform the other models. While our grid search for model 1 yielded no variability in three of the four optimized parameters (*init*_*KC*>*KC*_, *init*_*KC*>*DAN*_, $\alpha_{KC>MBON_{-}}$ , *α*_*KC*>*KC*_, Table 3), the hypersphere sampling revealed more optimal parameter combinations outside the boundaries of our grid search. The highest SOC performance found was 0.06, confirming the inferiority of this circuit.

A third approach to comparing the robustness between the different model circuits is to quantify how well they retain their FOC and SOC performance when variability is introduced into the connectivity matrix, the strength of the KC>MBON synapses, or the learning rate.

**FIGURE 5:**
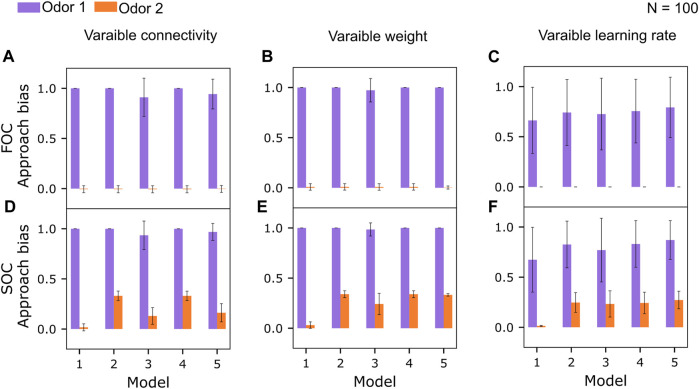
Robustness of the learning performance across variations of the KC>MBON connectivity. **(A)** Mean approach biases (error bars denote standard deviations, Eq. 1) to odors 1 and 2 after first-order conditioning (FOC) for *N* =100 model instances with a varying number of KC>MBON synapses, between 25% and 100%, drawn randomly from a uniform distribution. Responses to odor 3 are always 0 and not plotted. **(B)** Mean approach biases (Eq. 1) to odors 1 and 2 after first-order conditioning (FOC) for N = 100 model instances with a randomly chosen initial KC>MBON weights (full connectivity), varying ±5% around the default value (Table 1). **(C)** Mean approach biases (Eq. 1) to odors 1 and 2 after first-order conditioning (FOC) for N = 100 model instances with a KC>MBON^−^ learning rate, drawn from a uniform distribution ranging from the minimum to the maximum of learning rates tested (Table 3). **(D)** Mean approach biases (error bars denote standard deviations, Eq. 1) to odors 1 and 2 after second-order conditioning (SOC) for *N* =100 model instances with a varying number of KC>MBON synapses, between 25% and 100%, drawn randomly from a uniform distribution. **(E)** Mean approach biases (Eq. 1) to odors 1 and 2 after second-order conditioning (SOC) for N = 100 model instances with a randomly chosen initial KC>MBON weights (full connectivity), varying ±5% around the default value (Table 1). **(F)** Mean approach biases (Eq. 1) to odors 1 and 2 after second-order conditioning (SOC) for N = 100 model instances with a KC>MBON^−^ learning rate, drawn from a uniform distribution ranging from the minimum to the maximum of learning rates tested (Table 3).

We varied either the number of input KCs into each MBON (Figure 5A) or the strength of the synaptic connections while retaining full connectivity (Figure 5B) or the learning rate (Figure 5C) for FOC and ran the same experiments for SOC as well (Figures 5D–F).

We varied the number of KCs providing input to each MBON between 25% and 100% for each model instance (*N* = 100 model instances) while maintaining the magnitude of the individual weights (Table 1, $init_{KC>MBON_{+}}$ / $init_{KC>MBON_{-}}$ ). For each model instance, a random number of connections was drawn from a uniform distribution and applied to each MBON (MBON^+^, MBON^−^) to select the same amount of random connections to be active. While FOC performance remained very robust across all models 1–5 (Figure 5A), SOC performance was significantly impaired in model 1 and model 3 (Figure 5D), compared to SOC performance with full connectivity (Figure 3A).

Additionally, we evaluated the robustness of the learning performance when the strength of the KC>MBON weights was varied in a range of ±5% around their default weight (Table 1). Again, no significant differences were observed for FOC performance (Figure 5B). SOC performance was retained for models 2, 4, and 5 (Figure 5E) compared to the standard model with the same strength of all synaptic weights (Figure 3A).

Finally, varying the KC>MBON^−^ learning rate within the corridor provided by the minimum and maximum value of the parameter optimization (Table 3) (Figure 3C, F) showed overall increased variability in model FOC and SOC performance, while retaining equally relatively strong average SOC performance for models 2–5.

While all five circuit motifs were capable of FOC (Figure 2), model 1 performed very poorly at SOC compared to the other models (Figure 2). While model circuits 3–5 fulfill the criteria for SOC, they differ in their robustness to reward generalization and variations of their parameters. Model 2, which relies on plastic feed-forward input of KCs to the DAN, emerges as a promising candidate, in addition to the circuits that are already being explored (Rachad, 2023; Yamada et al., 2023).

## Discussion

While SOC as a phenomenon has been a target of insect learning experiments for a long time (Bitterman et al., 1983; Brembs and Heisenberg, 2001; Hussaini et al., 2007; Tabone and de Belle, 2011; König et al., 2019; Yamada et al., 2023), the discovery of the underlying circuit mechanisms is just at its beginning (Dylla et al., 2017; König et al., 2019; Rachad, 2023; Yamada et al., 2023). We used mechanistic models of different variations of a basic, abstract MB circuit inspired by *Drosophila melanogaster* and showed that different circuit motifs, based on either KC or MBON-driven DAN activation, can support SOC. In the following, we will discuss our results in light of experimental evidence for SOC in insects and the extent to which the MB anatomy supports the tested circuit motifs.

### Second-order conditioning in insect experiments and models

In second-order conditioning experiments, learning can be quantified using different measures. In honeybees (Bitterman et al., 1983; Hussaini et al., 2007), proboscis extension was measured as a response to conditioning with odors and sugar. Regardless of whether the number of SOC trials was equal to (Hussaini et al., 2007) or 50% of the number of FOC trials (Bitterman et al., 1983), the conditioned response acquired during FOC was stronger than that acquired during SOC. In the fly experiments, a combination of odor and electric shock (Tabone and de Belle, 2011), odor and sugar (Yamada et al., 2023), or odor and optogenetic DAN activation (Yamada et al., 2023) were used. The same duration of pairing an electric shock with odor 1 and pairing odor 1 with odor 2 during SOC yielded a stronger learning effect for FOC, compared to the SOC effect (Tabone and de Belle, 2011), as observed in bees (Bitterman et al., 1983; Hussaini et al., 2007). Yamada et al. (Yamada et al., 2023) used a protocol with longer FOC than SOC duration in an appetitive conditioning protocol. This led to similarly strong FOC and SOC effects, given a long enough FOC training duration.

Potential circuit mechanisms behind SOC were investigated in some studies conducted in *Drosophila* (König et al., 2019; Rachad, 2023; Yamada et al., 2023). Evidence for a mechanism based on MBON>DAN feedback comes from a study that used optogenetic silencing or activation of MBONs as an indirect punishment or reward signal for conditioning avoidance or approach of an odor, thereby circumpassing pairing of reinforcement and stimulus 1 during FOC (König et al., 2019). Yamada et al. (Yamada et al., 2023) also suggest an MBON>DAN pathway across two layers of interneurons as a mechanism for SOC. They showed that a presentation of an odor with optogenetic DAN activation can induce suppression of the response of a particular MBON (*α*1). Decreased activity of MBON-*α* could cause disinhibition of multiple pathways via two interneurons, leading to a net activation of DANs that encode reward during SOC. The circuit for the disinhibition of the DAN during SOC is closely related to the motif implemented in our model 4, which performs well at SOC but appears not to be very robust to reward generalization onto novel odors. Likewise, in an aversive conditioning paradigm, it was demonstrated that the output of a particular MBON, innervating the *γ*-lobe (MBON-*γ*1), is required during the SOC phase of the experiment to induce a learned valence of the second odor (Rachad, 2023). Similarly, a single MBON (MBON-*α*′2) innervating the *α*’/*β*′-lobes seems to play a similar role in these lobes (Rachad, 2023). In summary, all of these works demonstrate the importance of MBON output pathways (König et al., 2019; Rachad, 2023; Yamada et al., 2023) and seem to suggest MBON>DAN feedback as a prime candidate mechanism for SOC. For the sake of completeness, it has to be stated that each experiment targeted specific pathways and can not rule out the presence of different circuit motifs for SOC in other compartments.

Some recent models of the adult (Faghihi et al., 2017; Jiang and Litwin-Kumar 2021) or larval (Eschbach et al., 2020) *Drosophila* MB can accommodate SOC. They all suggest KC>MBON plasticity to learn stimulus 2 during SOC via MBON activity. Either in the form of net excitatory and inhibitory MBON>DAN feedback (Eschbach et al., 2020; Jiang and Litwin-Kumar 2021) or via direct modulation of the KC>MBON synapses by altered MBON activity (Faghihi et al., 2017). None of the models allow KC input to the DAN and thus do not test this alternative pathway.

Since some experimental and modeling studies have demonstrated or suggested competing circuit mechanisms for SOC, it seems likely that more than one mechanism exists within the highly connected structure of the MB. By exploring a variety of computationally feasible circuits in parallel, we wanted to provide a different perspective to integrate these opposing views.

### Anatomical evidence for the tested circuit motifs

To evaluate the biological plausibility of our tested circuit motifs, we next assessed which DAN-activation pathways are supported by anatomical evidence. KC>DAN synapses have been found both in larval (Eichler et al., 2017; Saumweber et al., 2018; Eschbach et al., 2020) and adult (Cervantes-Sandoval et al., 2017; Takemura et al., 2017; Scheffer et al., 2020) *Drosophila*. Since KCs are known to be cholinergic (Barnstedt et al., 2016) in the adult, it has been suggested that these connections are excitatory (Eichler et al., 2017) in the larva. This has also been confirmed in the adult, regarding the effect on a particular MBON (*α*2*α*′2) (Cervantes-Sandoval et al., 2017).

Direct and indirect connections between MBONs and DANs exist in the larva (Eichler et al., 2017; Eschbach et al., 2020) and the adult (Ichinose et al., 2015; Takemura et al., 2017) within and between compartments (Ichinose et al., 2015; Eichler et al., 2017; Takemura et al., 2017; Eschbach et al., 2020). In the larva, excitatory and inhibitory synapses have been observed (Eichler et al., 2017; Eschbach et al., 2020). In the adult *Drosophila* MB, different MBONs have been found to release excitatory or inhibitory transmitters (Aso et al., 2014b), supporting the assumption that here both de- and hyperpolarizing effects of MBONs on DANs exist.

Direct KC>KC synapses have been found in large numbers in the larva (Eichler et al., 2017). According to the transmitter released by KCs in the adult (Barnstedt et al., 2016), they have been speculated to be excitatory (Eichler et al., 2017). In the adult, KC>KC synapses have also been demonstrated (Takemura et al., 2017; Manoim et al., 2022). KCs have been shown to express both muscarinic (Bielopolski et al., 2019; Manoim et al., 2022) and nicotinic (Crocker et al., 2016; Croset, Treiber, and Waddell, 2018) receptors, the combination of which likely enables both inhibitory (Bielopolski et al., 2019; Manoim et al., 2022) and excitatory (Su and O’Dowd, 2003) synapses between them, rendering different interactions plausible.

All tested circuit motifs are sensitive to odor overlap among the KCs (Figure 3), demonstrating the importance of separable odor representations to avoid reward generalization. This hints at the importance of KC coding space, which some of the circuits would be more or less sensitive to, depending on the degree of odor specificity of their SOC mechanism (Figure 2). We based the size of the KC population on adult *Drosophila*, but as long as the three odors can be represented in a disjunct manner, the results (Figure 2) would not be affected. Interestingly, while SOC has been demonstrated in adult *Drosophila* (Brembs and Heisenberg, 2001; Tabone and de Belle, 2011; König et al., 2019; Rachad, 2023; Yamada et al., 2023) and other insects with larger KC population (Bitterman et al., 1983; Hussaini et al., 2007; Mizunami et al., 2009), it has never been observed in *Drosophila* larva, which might be due to its lack of KC coding space (Eichler et al., 2017).

### Limitations

Motivated by isolating SOC as the phenomenon of interest in our experiments, we decided to reduce our circuit implementations of computational motifs to their minimum and optimize their parameters only for FOC and SOC. This allowed us to determine which circuit motifs are the most efficient in computing SOC when optimized exclusively for this purpose. This approach neglects the surrounding network structures in the real insect MB and thus intentionally disregards other learning phenomena often addressed when studying the MB, such as prediction error, effects of stimulus exposure before learning, forgetting, or extinction. Both forgetting and extinction produce the same observable behavior in experiments, which is a decline in the response to repeated presentation of a sensory cue when reinforcement is omitted after conditioning. As opposed to forgetting, extinction is characterized by the possibility of recovery of the association after its temporary loss (Bouton, 2004), has been demonstrated in adult *Drosophila* (Hirano et al., 2016; Wang et al., 2019). To retain the association for recovery, extinction requires the formation of parallel memory traces for the acquisition and the decline of the association (Felsenberg et al., 2017; Felsenberg et al., 2018). The repeated presentation of stimulus 1 without reinforcement during SOC should lead to the extinction of the learned association between stimulus 1 and reinforcement. Across many trials, SOC and extinction learning should be competing phenomena, allowing SOC to occur only until the extinction process has abolished the odor approach bias. While some studies found a decline in the association between stimulus 1 and the reinforcement during SOC in honeybees (Bitterman et al., 1983; Hussaini et al., 2007), experiments in *Drosophila* reported no loss of the association between stimulus 1 and reinforcement during SOC (Tabone and de Belle, 2011; Yamada et al., 2023). For our *Drosophila*-inspired modeling approach, we thus defined no loss of odor 1 approach bias between the tests following FOC and SOC as a criterion for our parameter optimization. We did not include any homeostatic mechanism for forgetting in the models. The implementation of extinction would require the extension of the model with additional compartments for the encoding of an extinction trace in parallel to one that retains the learned association.

Additionally, the omission of mechanisms for long-term network stability in combination with the criterion of perfect odor 1 approach bias in both tests after FOC and SOC guarantee the complete downregulation of all KC>MBON^−^ synapses activated by odor 1 after three FOC trials of arbitrary duration would not allow for experiments with more trials.

Aside from the narrow applicability to different learning phenomena, which is the downside of our minimal circuit design, another limitation originates from the need to define the limits and the step size for the model parameter grid search. The success of it depends on selecting these limits and steps appropriately (Table 3). If ill-chosen, they could put individual models at a competitive disadvantage by not including or over-stepping their optimum.

### Outlook

We demonstrated that several circuit mechanisms are potential candidates for SOC. While they vary in computational efficiency and robustness, multiple models remain good candidates, compatible with our knowledge of the MB anatomy. A valuable next step would be to integrate them into more comprehensive MB models to test how they interact with other phenomena in learning. This could also be another angle to studying their robustness. Other models of learning in the MB have examined a variety of more complex aspects of learning, such as reinforcement expectation and prediction (Arena et al., 2015; Eschbach et al., 2020; Bennett, Philippides, and Nowotny, 2021; Jiang and Litwin-Kumar 2021; Springer and Nawrot, 2021; Zhao et al., 2021; Jürgensen et al., 2022), reinforcement generalization to other novel stimuli (Wessnitzer et al., 2012; Peng and Chittka, 2017; Rapp and Nawrot, 2020) as well as patterning tasks (Wessnitzer et al., 2012; Haenicke, 2015; Peng and Chittka, 2017), where combinations of stimuli are reinforced, while their components are not or *vice versa*. Spiking models, in particular, also allow the study of temporal dynamics in learning in the MB in experiments that feature delays or gaps between stimuli (Arena et al., 2015; Jürgensen et al., 2022). Especially interesting in this regard would be extinction, with its inherently interfering mechanism, which has been included in other models as well (Eschbach et al., 2020; Bennett, Philippides, and Nowotny, 2021; Jiang and Litwin-Kumar 2021; Springer and Nawrot, 2021). SOC relies on maintaining the stimulus 1 valence acquired during FOC throughout SOC, which drives DAN activity. Yet, the absence of reinforcement during SOC would trigger the extinction of this very valence. It seems possible that more than one of the circuit motifs could co-exist in different MB compartments. Ultimately, not all MB compartments might be involved in SOC (Jiang and Litwin-Kumar 2021; Yamada et al., 2023), but fulfill other roles in learning.

Computational models are a highly beneficial tool for investigating the circuitry underlying SOC. Experimental validations of theoretically proposed circuit motifs would close the loop between theoretical predictions and their experimental test. However, with the available genetic tools, it is currently impossible to solely manipulate KC>DAN or MBON>DAN synapses either on the pre or post-synaptic side without affecting output onto other or input from other neurons in the network. Therefore, an experimental test of our theoretical predictions is currently difficult to achieve, underlining the importance of computational modeling.

## Data Availability

The raw data supporting the conclusion of this article will be made available by the authors, without undue reservation.
